# Characterization of cDNAs Encoding Serine Proteases and Their Transcriptional Responses to Cry1Ab Protoxin in the Gut of *Ostrinia nubilalis* Larvae

**DOI:** 10.1371/journal.pone.0044090

**Published:** 2012-08-31

**Authors:** Jianxiu Yao, Lawrent L. Buschman, Brenda Oppert, Chitvan Khajuria, Kun Yan Zhu

**Affiliations:** 1 Department of Entomology, Kansas State University, Manhattan, Kansas, United States of America; 2 USDA Agricultural Research Service, Center for Grain & Animal Health Research, Manhattan, Kansas, United States of America; University of Kentucky, United States of America

## Abstract

Serine proteases, such as trypsin and chymotrypsin, are the primary digestive enzymes in lepidopteran larvae, and are also involved in *Bacillus thuringiensis* (Bt) protoxin activation and protoxin/toxin degradation. We isolated and sequenced 34 cDNAs putatively encoding trypsins, chymotrypsins and their homologs from the European corn borer (*Ostrinia nubilalis*) larval gut. Our analyses of the cDNA-deduced amino acid sequences indicated that 12 were putative trypsins, 12 were putative chymotrypsins, and the remaining 10 were trypsin and chymotrypsin homologs that lack one or more conserved residues of typical trypsins and chymotrypsins. Reverse transcription PCR analysis indicated that all genes were highly expressed in gut tissues, but one group of phylogenetically-related trypsin genes, *OnTry-G2*, was highly expressed in larval foregut and midgut, whereas another group, *OnTry-G3*, was highly expressed in the midgut and hindgut. Real-time quantitative PCR analysis indicated that several trypsin genes (*OnTry5* and *OnTry6*) were significantly up-regulated in the gut of third-instar larvae after feeding on Cry1Ab protoxin from 2 to 24 h, whereas one trypsin (*OnTry2*) was down-regulated at all time points. Four chymotrypsin and chymotrypsin homolog genes (*OnCTP2*, *OnCTP5*, *OnCTP12* and *OnCTP13*) were up-regulated at least 2-fold in the gut of the larvae after feeding on Cry1Ab protoxin for 24 h. Our data represent the first in-depth study of gut transcripts encoding expanded families of protease genes in *O. nubilalis* larvae and demonstrate differential expression of protease genes that may be related to Cry1Ab intoxication and/or resistance.

## Introduction

The European corn borer, *Ostrinia nubilalis*, is an important insect pest of corn, *Zea mays*, in the Midwest corn belt of the United States [1]. Currently, *O. nubilalis* is controlled mostly by transgenic corn expressing Cry1Ab and Cry1F toxins from *Bacillus thuringiensis* (Bt) [2] and this corn is commonly known as Bt corn. Although transgenic Bt corn is effective in controlling *O. nubilalis*, this technology would be threatened by populations of *O. nubilalis* resistant to transgenic corn [3]. At present, resistance management relies on the high dose/refuge strategy, with non-Bt corn planted in a refuge to conserve susceptible alleles in the field [4].

Cry protoxins are naturally occurring insecticidal proteins produced by *B. thuringiensis,* a soil inhabiting bacterium. The mode of action of Cry protoxins in susceptible insects includes solubilization of the crystalline protein, proteolytic processing of protoxin to activated toxin, and binding of activated toxin to midgut receptors. Any physiological modification to this cascade of events can result in a reduction in insect susceptibility to the toxin. For example, changes in the expression or type of proteolytic digestive enzymes, such as trypsin and chymotrypsin, can alter the toxicity of Bt toxins by reducing toxin solubility and reducing the activation of protoxin [5].

The lepidopteran larval gut represents a complex proteolytic environment containing serine proteases (trypsins, chymotrypsins, and elastases), aminopeptidases, and carboxypeptidases, involved in food digestion [6]. Trypsins can contribute up to 95% of the total digestive activity in the lepidopteran larval gut [7]. However, in other insects, such as the cockroach, some beetles, mosquito larvae, wasps and hornets, chymotrypsins are the major enzymes in protein digestion [8]. For example, chymotrypsins play the major role in proteolytic activity of the homopteran *Eurygaster integriceps* [9]. In lepidopterans, most trypsins and chymotrypsins have properties related to the extreme alkaline pH in the larval gut lumen [6].

The phenomenon of plant expressed protease inhibitors (PIs) to defend against feeding by phytophagous insects was described first in the 1950's [10] and has since been studied extensively. Transgenic plants have been developed with specifically enhanced PI expression to suppress phytophagous insect damage [11], [12]. However, insects can avoid PI toxicity by expressing proteases that are insensitive to host plant PIs [13], [14] or by increasing the proteolytic inactivation of PIs [15]. For example, Volpicella et al. [16] purified two different types of trypsins, HzTrypsin-C and HzTrypsin-S, from *Helicoverpa zea*. Both trypsins were involved in digesting plant material, but HzTrypsin-C was sensitive to plant inhibitors, whereas HzTrypsin-S was insensitive, suggesting that HzTrypsin-S evolved as a defense mechanism against plant PIs by the insect. Similarly, two genes encoding trypsins were identified from *S. frugiperda* [17]. One was constitutively expressed before or after exposure to soybean trypsin inhibitor (SBTI), and other was only induced after ingestion of SBTI. These studies suggest that the diversity of serine proteases in the insect gut is an adaptation against plant-produced PIs.

Many lepidopteran insects have expansions of genes that encode trypsins and chymotrypsins. Lepidopteran gene expansions have been found in *Helicoverpa zea* [13], *Manduca sexta* [18], *Choristoneura fumiferana* [19], *Plodia interpunctella* [20], *Helicoverpa armigera* [21], *Agrotis ipsilon* [14], [22], *Sesamia nonagrioides* [23], *Tineola bisselliella* [24], *Bombyx mori* [25], *Spodoptera litura* [26], *Bombyx mandarina* [27], *S. frugiperda* [28], *Mamestra configurata* [29], *O. nubilalis* [30], and *Heliothis virescens* [31]. Protease gene expansions are proposed to have occurred in response to the evolutionary selection pressure of plant inhibitors [32].

Serine proteases have been found to be associated with both crystal and spore formation in *B. thuringiensis* [33] and play key roles in activating and/or degrading Bt Cry-toxins [34]–[36]. For example, trypsin has been shown to generate an insecticidal toxin from a 130-kDa protoxin of Bt subsp. *kurstaki* HD-73 [34]. Seven specific cleavages are identified to occur in an ordered sequence starting at the C-terminus of the protoxin and proceeding toward the N-terminal region. In *P. interpunctella*, serine protease inhibitors can reduce gut protease activities and prevent the hydrolysis of the Bt protoxin Cry1Ac [35]. Furthermore, trypsin and chymotrysin are found to be involved in Cry1A protoxin activation in the same insect [36]. In *O. nubilalis*, we demonstrated that there was reduced trypsin activity in a Bt-resistant strain of *O. nubilalis* resulting in reduced Cry1Ab protoxin activation [37]. Further, we identified a transcript encoding trypsin (OnTry23) in this Bt-resistant strain with reduced expression compared with that of a susceptible strain [38].

**Table 1:** Primers used in RT-PCR and qPCR analyses of 34 putative trypsin and chymotrypsin and homolog genes in *O. nubilalis* larvae.

Gene name	Primer sequence (5′-3′)	Product size (bp)	Protease group
*OnTry1*	ATGCGTACCTTCATCGTTCTAC	116	Try-G2
	GCCATCTCAGGGTATTGGTTAATG		
*OnTry4*	ACCTGTCCATCATCCGAACC	157	
	TCAGACGACGATCCTCCTTG		
*OnTry5*	GGACAGTTCTCTGAGCAGTTAC	109	
	ACAGCATGTTGTCAGTGATGG		
*OnTry6*	ATTCTCAACAACAGGGCTATTTTG	148	
	TGTAGTCAGGGTGGTTAATGATTC		
*OnTry7*	CATCACGGAGAACATGCTTTG	158	
	CGTTGACACCAGGGAAGAAG		
*OnTry8*	TGTTTCATCGGTACTGTCACTG	193	
	GAGGATCACTCGTCTGTTAAGG		
*OnTry9*	GAGTGGGGTCTTCCTTCAGG	105	
	CAGCAATGTCGTTGTTAAGCG		
*OnTry14*	GCATCATACCCGTCACATCTAC	148	
	GTGAAGTTGCCGTACTGAGTC		
*OnTry11*	CTGGTGGAGTTATTGCCTACG	133	Try-G1
	GTGGTTTGCTGGATGGATGG		
*OnTry3*	TCAGACTGGTCACCCCTTTC	196	
	TCACGGCATAGGTTGTTGTTG		
*OnTry22*	ATGGCGTCCTCGTTGGTG	82	
	TGGTGCCTCCCACAATGC		
*OnTry23*	GAGACCACCATCAACGAGTATC	92	
	ATGTTAGCAGCACACGACTG		
*OnTry21*	CAACTACGCCACTCTCTCATC	175	
	GACGCCAGGGAAGCCATC		
*OnTry10*	CACAAAGTCCTGGAGGAAGATTC	103	Ungrouped*
	GTTCACGCCTGTCTGTTGC		
*OnTry2*	CACAAAGTCCTGGAGGAAGATTC	125	
	GTTCACGCCTGTCTGTTGC		
*OnTry12*	GCCAGCATTACACCTTCCG	128	Try-G4
	TCGCAGTTCTCGTAGTAAGAC		
*OnTry13*	CATCATCATCCACCCAGACTATG	187	
	GGCACCGTCCTCTTCCTC		
*OnCTP1*	ACCTGCCTACCAGCGTTTC	112	CTP-G1
	CCGAAGCCTGAAGCAATAGC		
*OnCTP4*	GCTGGTTCCCTCTACTGGTC	79	
	GAGATGGTGTTGGAGAAGGC		
*OnCTP5*	TTGCGGGATACGGGAAGAC	85	
	GGAGATTGACCGAGTGGAGAG		
*OnCTP3*	TGTGATCCAGCCCATCTCTC	95	
	CAGAAGTGCGTCCGAATCC		
*OnCTP17*	CCCCTTCGTCCACGCTAG	123	
	GTCACACCAACCAAGAGTCTC		
*OnCTP7*	TTGCGGGATACGGGAAGAC	75	
	GGAGATTGACCGAGTGGAGAG		
*OnCTP6*	TCGGGACAACTGGTCTAGC	114	
	CGCACTCGTCGTTAGGTATC		
*OnCTP8*	GCCGCTGGATTTGGAAAGAC	135	
	GAGGGTGCTCGGGAATACG		
*OnCTP9*	TCAGTGGAACCCGTGGAAC	94	
	CAGTGCGATTGGTTGGATGG		
*OnCTP10*	CCTACTGAGGATGCGAATAACG	96	
	TGGGTTGGCTGGGTTTGG		
*OnCTP11*	ATAGAGCACCCGAATTACAACG	123	
	GTAGGTTTGCGAGCCAGTG		
*OnCTP12*	CCAGATCAACCGCATCGC	110	
	TTCCTGAAGAAGCCAGTAAACC		
*OnCTP2*	GAGGAGGGCACGGACTTC	106	
	TTCCTGTGTTCAAGGTGATGAC		
*OnCTP13*	TCTTCTCAACCACGACTTAACG	117	
	ATTACTTGAAGCGACACAAATCTG		
*OnCTP14*	GTAAGACTGGTCGGTGGTAAAG	149	Ungrouped*
	TCGGCTCCAAGAACACAATG		
*OnCTP15*	CCATAGGTGAGAAGGAATGTGC	164	CTP-G2
	GTTGGTCTTCAGCGATACTAGAG		
*OnCTP16*	TCCTCGCCTGTGGTGTTC	156	
	GATGGTGGTCACGGTCAAC		

* These deduced protein sequences have low bootstrap values (<75) and are not included in groups.

In this study, we sequenced and characterized 34 full-length transcripts encoding putative trypsins, chymotrypsins, and their homologs from an *O. nubilalis* gut specific cDNA library. Transcripts were systemically analyzed in *O. nubilalis* larvae for tissue-specific expression patterns and transcriptional responses after ingestion of Cry1Ab protoxin. These studies provide the first comprehensive data set of protease genes in the gut of *O. nubilalis* larvae and the first insights into how Cry toxins may influence their transcription.

**Table 2:** Characteristics of 12 putative trypsins and four homologs deduced from full-length cDNAs derived from the larval midgut of *O. nubilalis*.

Gene name	Number of amino acid residues	Predicted molecular mass (kDa)	Predicted isoelectric point (pI)	NCBI accession number
*OnTry1*	256	27.3	8.99	AY953063.1
*OnTry2*	395	42.1	7.39	AY513652.2
*OnTry3*	263	28.2	5.47	AY953064.1
*OnTry4*	257	28.0	9.19	JQ904122
*OnTry5*	258	27.4	8.55	JQ904123
*OnTry6*	263	28.7	8.53	JQ904124
*OnTry7*	258	28.1	6.04	JQ904125
*OnTry8*	257	27.3	9.45	JQ904126
*OnTry9*	261	27.7	6.70	JQ904127
*OnTry10*	321	34.7	8.27	JQ904128
*OnTry11*	262	28.8	4.48	JQ904129
*OnTry12*	265	28.0	4.61	JQ904130
*OnTry13*	273	29.7	4.49	JQ904131
*OnTry21*	256	25.9	4.78	EU673450.1
*OnTry22*	257	27.4	7.64	EU673451.1
*OnTry23*	257	27.4	6.21	

**Table 3:** Characteristics of 12 putative chymotrypsins and four homologs deduced from full-length cDNAs derived from the larval midgut of *O. nubilalis*.

Gene name	Number of amino acid residues	Predicted molecular mass (kDa)	Predicted isoelectric point (pI)	NCBI accession number
*OnCTP1*	289	30.6	7.66	AY953053
*OnCTP2*	280	29.3	9.12	AY953056
*OnCTP3*	287	29.6	7.67	EU673454
*OnCTP4*	294	30.9	5.48	JQ904133
*OnCTP5*	297	31.4	5.51	JQ904134
*OnCTP6*	284	30.1	8.51	JQ904135
*OnCTP7*	283	29.7	8.79	JQ904136
*OnCTP8*	298	31.8	6.36	JQ904137
*OnCTP9*	296	31.5	7.04	JQ904138
*OnCTP10*	281	30.9	5.36	JQ904139
*OnCTP11*	301	32.1	9.16	JQ904140
*OnCTP12*	280	29.6	9.71	JQ904141
*OnCTP13*	313	33.8	8.75	JQ904142
*OnCTP14*	246	27.3	5.97	JQ904143
*OnCTP15*	278	30.5	5.87	JQ904144
*OnCTP16*	281	29.5	7.14	JQ904145

## Materials and Methods

### Insect Rearing

The Bt-susceptible strain (Lee) of *O. nubilalis* was obtained from French Agricultural Research Inc. (Lamberton, MN). Larvae were reared under long-day conditions (L:D = 16:8) at 26°C on artificial diet (Bio-Serv. Inc. Frenchtown, NJ). Adults were reared in metal net cages lined with wax paper under long-day conditions with 70% humidity, and were routinely fed 2% sucrose water to provide supplementary nutrition. Eggs were collected from the wax paper every day and kept in insect rearing cups with high humidity (≥80%) until hatching. Newly hatched larvae were immediately transferred to artificial diet and reared to third or fifth instar for testing. The larval developmental stage was determined by moving them to a new rearing dish after each molt.

**Figure 1:**
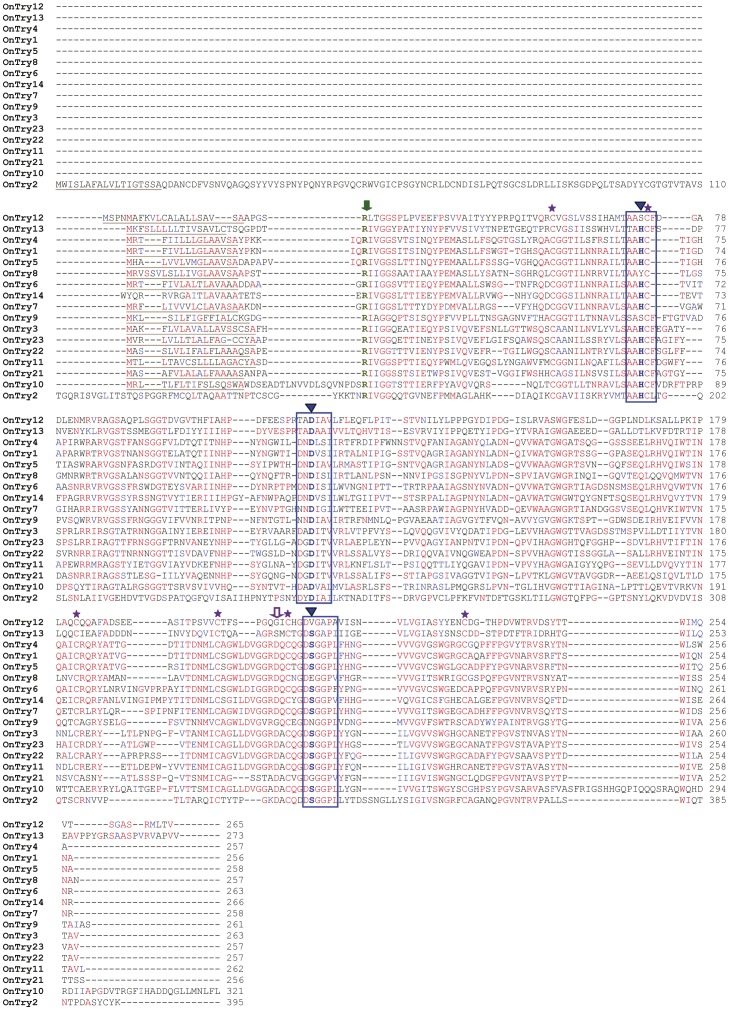
Multiple alignments of 17 deduced amino acid sequences of putative trypsins and trypsin homologs from *O. nubilalis* larvae using Clustal W2. The predicted signal peptide is underlined in brown; the catalytic triads and conserved regions are boxed in blue; the conserved catalytic triads are marked with blue arrows at the top; the autocatalytic site is marked with green arrow at the top; the conserved residue in the S1 pocket (or trypsin-determination residue) is marked with purple arrow at the top; and the six cysteine residues were marked with purple stars at the top.

**Figure 2:**
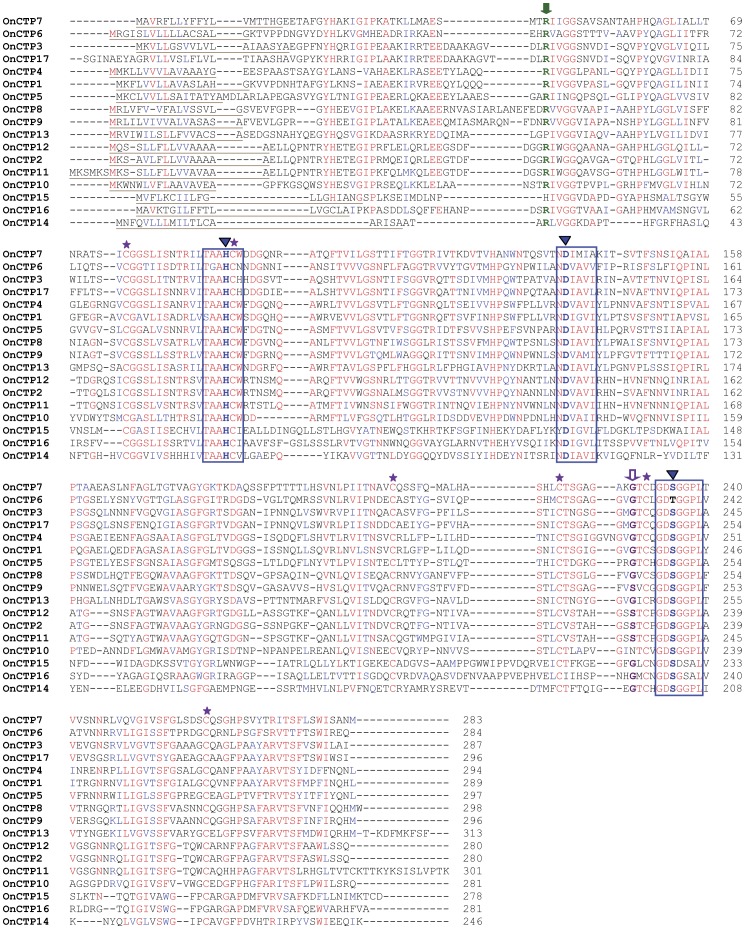
Multiple alignments of 17 deduced amino acid sequences of putative chymotrypsins and chymotrypsin homologs from *O. nubilalis* larvae using Clustal W2. The predicted signal peptide is underlined in brown; the catalytic triads and conserved regions are boxed in blue; the conserved catalytic triads are marked with blue arrows at the top; the autocatalytic site is marked with green arrow at the top; the conserved residue in the S1 pocket is marked with purple arrow at the top; and the six cysteine residues were marked with purple stars at the top.

### Identification and Sequencing of cDNAs

The cDNA sequences for 34 putative trypsins, chymotrypsins and their homologs were identified from sequences obtained from an *O. nubilalis* gut-specific cDNA library [39] by Blast2GO analysis. Ten were full-length cDNAs, and the remaining were amplified from partial cDNA sequences, using 5′ and 3′ rapid amplification of cDNA end (RACE) (Clontech SMART^TM^ RACE cDNA amplification kit, Mountain View, CA). Total RNA samples from the gut tissues of fifth instar larvae were used for cDNA synthesis, and RACE was conducted with one gene specific primer and another 5′ or 3′ universal primer using the touchdown PCR programs. The specific PCR product was sub-cloned into plasmid T-vector and sequenced using an ABI 3700 DNA sequencer at the Kansas State University DNA Sequencing Facility (Manhattan, KS). cDNA sequences were confirmed by gene specific primer pairs (Table 1) in both directions.

**Table 4:** Designations and key amino acid residues used to distinguish putative trypsins and chymotrypsins and their corresponding homologs from the larval gut of *Ostrinia nubilalis*.

Designation of serine proteases		Protease name	N-terminal activation residue (Arg or Lys)	Catalytic triad residues (His/Asp/Ser)	Number of Cys for disulfide bonds	S1 pocket
Trypsin	Putative	OnTry1	Arg	His/Asp/Ser	6	Asp
		OnTry2	Arg	His/Asp/Ser	6	Asp
		OnTry3	Arg	His/Asp/Ser	6	Asp
		OnTry4	Arg	His/Asp/Ser	6	Asp
		OnTry5	Arg	His/Asp/Ser	6	Asp
		OnTry6	Arg	His/Asp/Ser	6	Asp
		OnTry7	Arg	His/Asp/Ser	6	Asp
		OnTry10	Arg	His/Asp/Ser	6	Asp
		OnTry11	Arg	His/Asp/Ser	6	Asp
		OnTry14*	Arg	His/Asp/Ser	6	Asp
		OnTry22	Arg	His/Asp/Ser	6	Asp
		OnTry23	Arg	His/Asp/Ser	6	Asp
	Homolog	OnTry8	Arg	Tyr/Asp/Glu	6	Asp
		OnTry9	Arg	Ser/Asp/Asn	6	Gly
		OnTry12	Arg	Ser/Asp/Val	6	Gly
		OnTry13	Arg	His/Asp/Ser	6	Ser
		OnTry21	Arg	His/Asp/Gly	6	Asp
Chymotrypsin	Putative	OnCTP1	Arg	His/Asp/Ser	6	Gly
		OnCTP2	Arg	His/Asp/Ser	6	Ser
		OnCTP3	Arg	His/Asp/Ser	6	Gly
		OnCTP4	Arg	His/Asp/Ser	6	Gly
		OnCTP5	Arg	His/Asp/Ser	6	Gly
		OnCTP7	Arg	His/Asp/Ser	6	Gly
		OnCTP8	Arg	His/Asp/Ser	6	Gly
		OnCTP9	Arg	His/Asp/Ser	6	Ser
		OnCTP11	Arg	His/Asp/Ser	6	Ser
		OnCTP12	Arg	His/Asp/Ser	6	Ser
		OnCTP14	Arg	His/Asp/Ser	6	Gly
		OnCTP17*	Arg	His/Asp/Ser	6	Gly
	Homolog	OnCTP6	Arg	His/Asp/Thr	6	Gly
		OnCTP10	Arg	His/Asp/Ser	6	Asn
		OnCTP13	Pro	His/Asp/Ser	6	Gly
		OnCTP15	His	His/Asp/Ser	6	Gly
		OnCTP16	Arg	His/Asp/Ser	5	Gly

* OnTry14 and OnCTP17 are derived from partial cDNA sequences.

### Analysis of cDNA and Deduced Amino Acid Sequences

Homology analyses of cDNAs were performed by Blast. Amino acid sequences were deduced from cDNA sequences using the online translation tool from ExPASy [40]. Signal peptide sequences were predicted by SignalP [41]. Multiple sequence alignments of cDNA sequences from *O. nubilalis* were performed with published sequences from *B. mori*, *B. mandarina*, *A. ipsilon*, *S. frugiperda*, *M. configurata*, *S. litura*, *M. sexta*, *Ostrinia furnacalis*, *S. nonagrioides*, *Galleria mellonella*, *P. interpunctella*, *H. zea*, *H. armigera*, *Helicoverpa punctigera, C. fumiferana*, *Lacanobia oleracea*, and *H. virescens* using ClustalW2 [42]. GenBank accession numbers of these sequences are provided in the results section. Dendrograms were constructed according to the neighbor-joining method and confidence bootstrap values were determined using bootstrap analysis with the separation supported (5000 of 5000 bootstrap), re-sampling steps using MEGA 4 [43].

**Figure 3:**
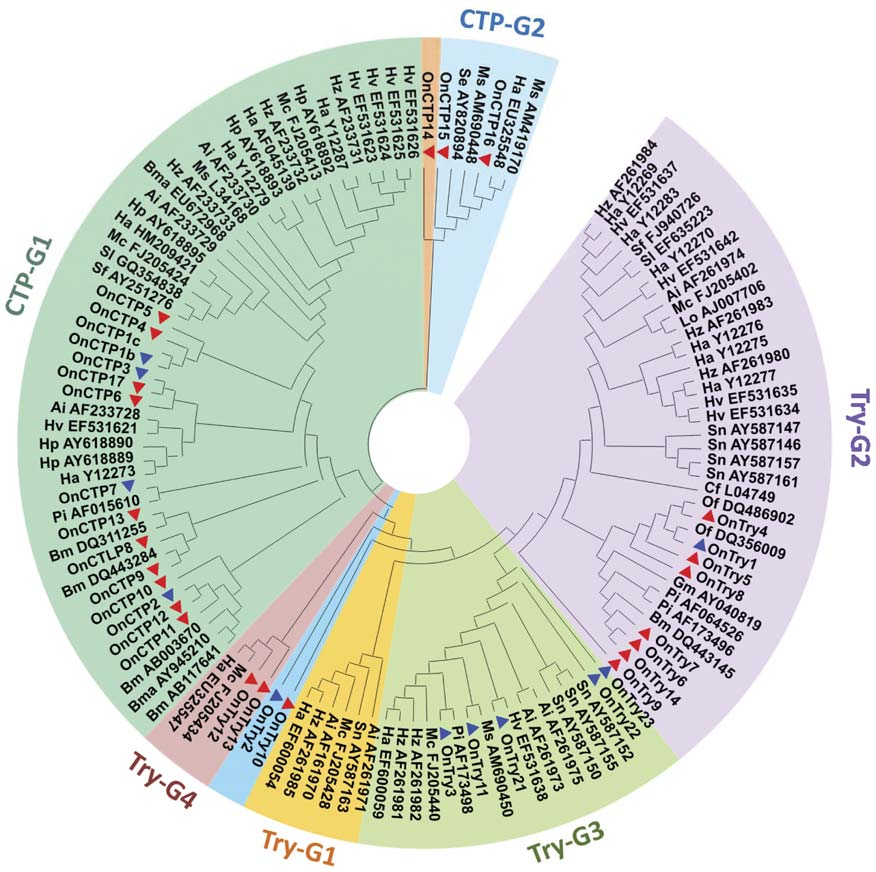
Phylogenetic analysis of amino acid sequences of 34 putative serine proteases from *O. nubilalis* with serine proteases from *P. interpunctella*, *C. fumiferana*, *H. armigera*, *M. sexta*, *B. mori*, *A. ipsilon*, *S. nonagrioides*, *S. frugiperda, S. litura*, *H. zea, B. mandarina, H. punctigera*, *M. configurata*, *S. exigua*, *L. oleracea*, *G. mellonella*, *O. furnacalis*. Bootstrap values are obtained by neighbor-joining method using 5000 replications. The blue “▴” indicated the trypsin genes that have already been submitted to the NCBI database; and the red “▴” indicates new cDNA sequences that were revealed from this study. The GenBank accession numbers of the sequences used in this analysis are listed in Table 2, 3, and 4.

### Analysis of Tissue-Dependent Gene Expression Patterns

Eight different tissues were dissected from fifth instar larvae, including foregut, hindgut, midgut, haemolymph, Malpighian tubules, carcass, fat bodies and silk gland. The tissue samples from five larvae were pooled and homogenized in 500 µl Trizol reagent (Invitrogen, Carlsbad, CA) for total RNA extraction and analyzed by NanoDrop 2000 spectrophotometer (Thermo Fisher Scientific, Waltham, MA) at 260 nm. First strand cDNA was synthesized from 1 µg of total RNA using a cDNA synthesis kit (Fermentas, Glen Burnie, MD). The gene specific primers were designed using Beacon Designer 7 (Primer Biosoft, Palo Alto, CA) (Table 1). Subsequently, reverse transcription (RT)-PCR was performed using 25 ng cDNA as template with 25 cycles of 95°C for 30 s, 56°C for 30 s and 72°C for 30 s. *O. nubilalis* ribosomal protein S3 (*OnRps3*) was used as a reference gene to detect the relative expression of 34 putative trypsin, chymotrypsin and homologous genes. Ten microliters of each PCR product were loaded onto 1.5% agarose gels containing ethidium bromide to examine tissue-specific expression patterns.

**Table 5:** GenBank accession numbers for other insect trypsins, chymotrypsins, and homologs that were used in phylogenetic analysis.

Organisms	GenBank accession
*Bombyx mori*	DQ443145, AB117641, AB003670, DQ443284, DQ311255
*Agrotis ipsilon*	AF261974, AF261975, AF261973, AF261971 AF161970, AF233730, AF233729, AF233728
*Spodoptera frugiperda*	FJ940726, AY251276
*Mamestra configurata*	FJ205402, FJ205440, FJ205428, FJ205434, FJ205424, FJ205413,
*Spodoptera litura*	EF635223, GQ354838,
*Spodoptera exigua*	AY820894
*Manduca sexta*	AM690450, L34168, AM419170, AM690448, AM690449
*Ostrinia furnacalis*	DQ356009, DQ486902
*Sesamia nonagriorides*	AY587147, AY587146, AY587157, AY587161, AY587152, AY587155, AY587150, AY587163,
*Ostrinia nubilalis*	AY953063, AY513650, EU673451, EU673450, AY953064, AY513652, AY953056, EU673454, AY953053, AY953054,
*Galleria mellonella*	AY040819
*Plodia interpunctella*	AF064526, AF173496, AF015610, AF173498
*Helicoverpa zea*	AF261984, AF261983, AF261980, AF261981, AF261982, AF261985, AF233731, AF233733, AF233732
*Helicoverpa armigera*	Y12269, Y12283, Y12270, Y12276, Y12275, Y12277, EF600059, EF600054, EU325547, EU325548, Y12287, AF045139, Y12279, HM209421, Y12273,
*Choristoneura fumiferana*	L04749
*Lacanobia oleracea*	AJ007706
*Heliothis virescens*	EF531637, EF531642, EF531635, EF531634, EF531638, EF531621, EF531624, EF531625, EF531623, EF531626,
*Bombyx mandarina*	AY945210, EU672968
*Helicoverpa punctigera*	AY618890, AY618892, AY618893, AY618895, AY618889

### Determination of Median Lethal Concentration of Cry1Ab Protoxin

Cry1Ab protoxin was prepared from *Escherichia coli* (strain ECE54) expressing the *cry1Ab* gene, based on the method previously described [44], and stored at −80°C as a suspension. The bacterial strain was provided by the *Bacillus* Genetic Stock Center, Ohio State University (Columbus, OH).

**Figure 4:**
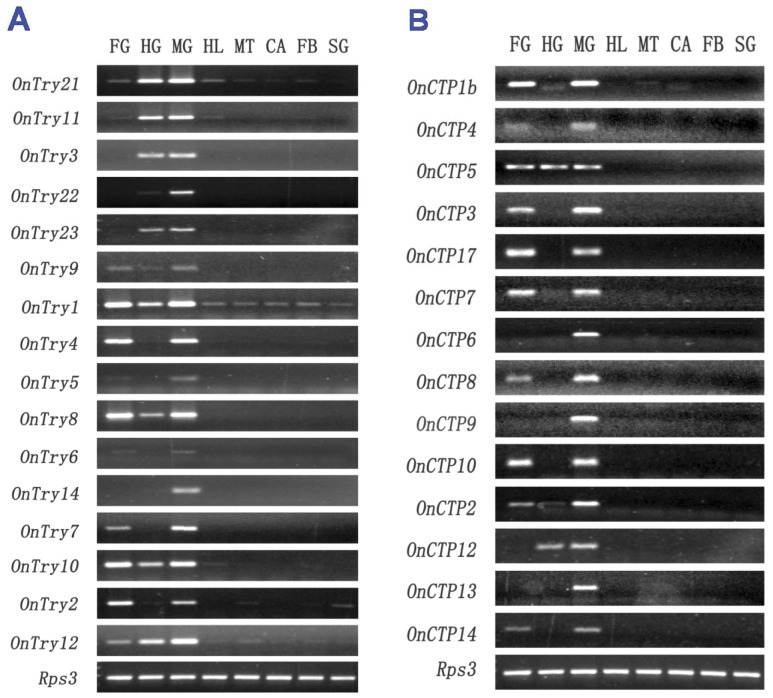
Expression of 31 serine protease transcripts in eight different tissues including foregut (FG), hindgut (HG), midgut (MG), haemolymph (HL), Malpighian tubules (MT), carcass (CA), fat bodies (FB) and silk gland (SG) from *O. nubilalis* larvae. The expression levels of *OnTry13*, *OnCTP15* and *OnCTP11* were too low to be detected by RT-PCR. *O. nubilalis* ribosomal protein S3 (*OnRps3*) was used as a reference gene.

The medial lethal concentration (LC_50_) of Cry1Ab protoxin was determined in a 7-d bioassay at room temperature. In this assay, third instar larvae were starved for 24 h, and larvae were fed artificial diet containing increasing concentrations of Cry1Ab protoxin (0, 0.04, 0.20, 1.0, 5.0, 25 µg/ml), with six biological replicates. Fresh artificial diet containing Cry1Ab protoxin was replaced every other day, and surviving individuals were recorded every day. The bioassay data were analyzed by probit regression using PROC GLM procedure [45]. After 7 d, the LC_50_ of third instar *O. nubilalis* larvae was 0.25 µg/ml (95% CI = 0.14–0.33 µg/ml). Mortality was not observed in control larvae fed artificial diet only.

**Figure 5:**
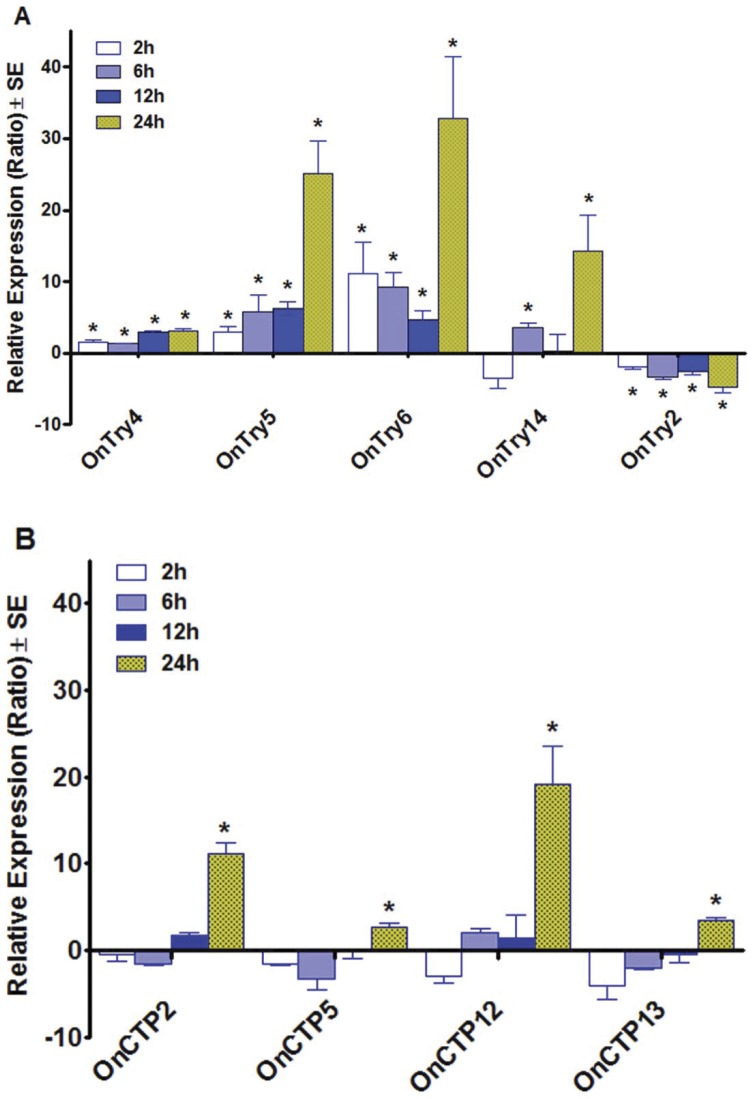
Relative expression ratios of putative trypsin and chymotrypsin transcripts in the gut of *O. nubilalis* early third instar larvae fed Cry1Ab protoxin at the LC_50_ concentration relative to larvae fed control diet at four different feeding periods. The asterisk “*” indicates that the transcript expression was significantly different at that feeding period (*P*<0.05), whereas the positive or negative values of each column indicates up- or down-regulation, respectively.

### Analysis of Transcriptional Response to Cry1Ab Protoxin Exposures

Early third instar *O. nubilalis* larvae were starved for 24 h at 26°C and were individually fed artificial diet containing either Cry1Ab protoxin (0.25 µg/ml) or no protoxin (control) for 2, 6, 12 and 24 h. Although this concentration of Cry1Ab was the LC_50_ for third instar larvae of *O. nubilalis*, it was determined based on a 7-d bioassay. At the same concentration of Cry1Ab, no mortality was observed after 24 h, but larvae were noticeably affected. The treatment was carried out with three biological replicates, each with five individuals. After the designated time, gut tissues were dissected, pooled for each treatment, and homogenized in Trizol reagent for total RNA extraction. First strand cDNA of each replicate was synthesized from 1 µg total RNA using a cDNA synthesis kit (Fermentas). Gene specific primer pairs (Table 1) were used in real-time quantitative PCR (qPCR) with 1 µl cDNA (1:25 dilution with ≈2 ng) as template in a 25 µl reaction (SYBR Green, Fermentas) with two technical replicates. qPCR was performed with 40 cycles of 95°C for 30 s, 56°C for 30 s, and 72°C for 30 s using a Bio-Rad IQ thermocycler (Bio-Rad Laboratories Inc. Hercules, CA). Relative expression differences were normalized to the *OnRps3* transcript level and analyzed using the comparative C_t_ method (ΔC_t_) [46]. Significance in the differential expression of transcripts from *O. nubilalis* larvae with or without exposure to Cry1Ab was determined by student *t*-test.

## Results

### Analysis of cDNA and Deduced Amino Acid Sequences

A total of 34 putative protease transcripts were identified from our *O. nubilalis* gut-specific cDNA library. cDNA sequences were further amplified using 5′ and 3′ RACE and confirmed by sequencing in both directions. Except for *OnTry14* and *OnCTP17* lacking a few nucleotides, the open reading frame of 32 full-length cDNA sequences ranged from 738 to 1185 bp. The cDNAs of *OnTry1*, *OnTry2*, *OnTry3*, *OnTry21*, *OnTry22*, *OnTry23*, *OnCTP1*, *OnCTP2* and *OnCTP3* were identical to those at NCBI [30], [38] (Tables 2 and 3), and the remaining 25 transcripts were discovered in this study. Among the 34 deduced amino acid sequences, 12 were putative trypsins, 12 were putative chymotrypsins, and the remaining 10 were trypsin or chymotrypsin homologs. The putative trypsins and chymotrypsins have typical conserved sequence motifs of trypsin and chymotrypsin, including N-terminal putative activation residues (Arg or Lys) for cleavage and formation of mature trypsins and chymotrypsins, three catalytic triad residues (His/Asp/Ser), and six Cys that form three disulfide bonds to maintain tertiary structure (Figs. 1 and 2, Table 4) [47]. *O. nubilalis* trypsins and chymotrypsins have an S1 binding pocket similar in primary sequence and tertiary structures to other similar proteases, and the substrate binding pocket has a negatively charged Asp in trypsin and a polar Ser in chymotrypsin [48]. Among 12 putative *O. nubilalis* chymotrypsins, OnCTP2, OnCTP9, OnCTP11, and OnCTP12 had the characteristic Ser in the S1 pocket, whereas the remaining 8 putative chymotrypsins had a Gly substitution (Fig. 2 and Table 4).

Five trypsin-like sequences (OnTry8, OnTry9, OnTry12, OnTry13, and OnTry21) and five chymotrypsin-like sequences (OnCTP6, OnCTP10, OnCTP13, OnCTP15 and OnCTP16) were designated homologs because the typical conserved sequence motifs of trypsin and chymotrypsin were substituted by other amino acid residues (Table 4, Figs. 1 and 2). These substitutions included N-terminal putative activation residues (Arg or Lys) replaced by Pro in OnCTP13 and His in OnCTP15; the conserved catalytic residues (His/Asp/Ser) were substituted by His/Asp/Val in OnTry12, Try/Asp/Glu in OnTry8, Ser/Asp/Asn in OnTry9, His/Asp/Gly in OnTry21 and His/Asp/Thr in OnCTP6; one cysteine residue was missing in OnCTP16; substrate binding pockets of trypsin (Asp) and chymotrypsin (Ser/Gly) were changed to Ser in OnTry13 and Asn in OnCTP10. The MEROPS databases classify enzymes with such substitutions as trypsin or chymotrypsin homologs [49].

### Comparison of Deduced Amino Acid Sequences from *O. nubilalis* and Other Lepidopteran Insects

The deduced amino acid sequences of 34 putative trypsins, chymotrypsins and their homologs, and those from other lepidopteran insects (Table 5), were used to construct a phylogenetic tree (Fig. 3). The neighbor-joining method was applied to the predicted amino acid sequence identity alignments to classify sequences into six groups (bootstrap value ≥73) with four trypsin groups (Try-G1, Try-G2, Try-G3 and Try-G4) and two chymotrypsin groups (CTP-G1 and CTP-G2). OnTry10, OnTry2 and OnCTP14 were not within these groups because of the lower bootstrap values (≤50). Trypsin homologs OnTry12 and OnTry13, grouped in Try-G4, and chymotrypsin homologs OnCTP15 and OnCTP16, grouped in CTP-G2, were the only *O. nubilalis* sequences within these groups and may have novel functions.

### Tissue-Dependent Gene Expression Profiles

Expression patterns of 34 *O. nubilalis* trypsin, chymotrypsin and their homolog transcripts were analyzed by RT-PCR in eight different tissues, including foregut, hindgut, midgut, haemolymph, Malpighian tubules, carcass, fat bodies and silk gland (Fig. 4). The expression of putative chymotrypsin (*OnCTP11*), chymotrypsin homolog (*OnCTP15*), and trypsin homolog (*OnTry13*) transcripts was too low to be detected by RT-PCR. However, the remaining transcripts were highly expressed in the gut. Transcripts encoding the putative trypsin and trypsin homologs of the Try-G3 group (*OnTry11*, *OnTry3*, *OnTry23*, *OnTry22* and *OnTry21*) were highly expressed in the midgut and hindgut (Fig. 4A). However, trypsin and trypsin homolog transcripts from the Try-G2 group (*OnTry1*, *OnTry4*, *OnTry5*, *OnTry6*, *OnTry7*, *OnTry8*, *OnTry14* and *OnTry9*) were highly expressed in the foregut and midgut. These data further support the phylogenetic grouping of the two main branches (Try-G2, Try-G3) and suggest that genes encoding these transcripts are localized in regions of the gut. *OnTry1* and *OnTry8* were also expressed in other tissues, including silk gland, fat bodies, and Malpighian tubules.

Of the 15 putative chymotrypsin and chymotrypsin homolog transcripts, nine were expressed in the foregut and midgut (*OnCTP1*, *OnCTP3*, *OnCTP4*, *OnCTP7*, *OnCTP8*, *OnCTP10*, *OnCTP14*, *OnCTP16*, and *OnCTP17*) (Fig. 4B). Only *OnCTP12* was expressed in both the midgut and hindgut, but not in the foregut. Two putative chymotrypsin transcripts (*OnCTP2* and *OnCTP5*) were expressed in all three gut sections; three (*OnCTP6*, *OnCTP9*, and *OnCTP13*) were midgut-specific genes. The expression of all putative chymotrypsin and chymotrypsin homolog transcripts could not be detected in other tissues by RT-PCR.

### Transcriptional Response to Cry1Ab Protoxin Exposures

The expression profiles of *O. nubilalis* trypsin, chymotrypsin and homolog transcripts were analyzed for third instar larvae fed artificial diet with and without Cry1Ab protoxin for four different feeding periods (2, 6, 12, and 24 h; Fig. 5). The expression levels of four putative trypsin transcripts, *OnTry4*, *OnTry5*, *OnTry6* and *OnTry14*, were correlated to increasing protoxin exposure (Fig. 5A). However, the expression of *OnTry2* was down-regulated at all Cry1Ab protoxin exposure time points. Expression of all other *O. nubilalis* trypsin transcripts was not affected by the ingestion of Cry1Ab protoxin (data not shown). The expression of all 17 putative chymotrypsin, and chymotrypsin homolog transcripts were not significantly affected by exposure to Cry1Ab protoxin for 2 to 12 h (data not shown). However, transcripts encoding three putative chymotrypsin (*OnCTP2*, *OnCTP5*, *OnCTP12*) and one chymotrypsin homolog (*OnCTP13*) were up-regulated at least 2-fold (*P*<0.05) after 24 h of exposure to Cry1Ab protoxin (Fig. 5B).

## Discussion

We sequenced and characterized 34 full-length transcripts encoding putative trypsins, chymotrypsins, and their homologs from an *O. nubilalis* gut specific cDNA library. This study represents the most comprehensive data to date on digestive proteases in an economically-important corn pest. We identified five trypsin-like transcripts including OnTry8, OnTry 9, OnTry21, OnTry12, and OnTry13. Because the deduced amino acid sequences of these transcripts lack conserved residues critical to proteolytic activity, they are referred to as trypsin homologs. Based on phylogenetic analysis, they were grouped in Try-G2 (OnTry8 and OnTry9), Try-G1 (OnTry21) and Try-G4 (OnTry12 and OnTry13), suggesting they represent gene families that may have arisen from mutation events that have been described in other insect species [50].

Theoretically, the putative chymotrypsin transcripts in the CTP-G1 group should have predicted proteins that contain conserved catalytic triads (His, Asp and Ser), six Cys residues forming disulfide bridges, conserved activation sites at the N-terminal of the mature enzyme, and a conserved serine in the S1 pocket. However, only four putative chymotrypsins (OnCTP2, OnCTP9, OnCTP11 and OnCTP12) have the conserved serine, and the remaining putative chymotrypsins have a Gly/Ser substitution in the S1 pocket. Loop regions adjacent to binding pockets of trypsin and chymotrypsin proteins have significant effects on enzyme activity, substrate specificity, and substrate binding specificity [51]. Loop structures adjacent to the substrate binding pocket have a negatively charged Asp in trypsin and a polar Ser in chymotrypsin [48]. Chymotrypsins have amino acids that allow for more flexibility in the loop regions than do trypsins to accommodate substrates with hydrophobic residues. In *M. sexta*, chymotryspins were demonstrated to cleave proteins on the carboxyl side of Tyr, Phe, Trp, His, and Thr [52]. This Gly/Ser substiution in the S1 pocket has been found in other insect proteases and has presumably minor effects on substrate interactions [53].

OnTry12 and OnTry13 are trypsin homologs and have orthologs in *M. configurata* (accession No: FJ205434) and *H. armigera* (accession No: EU325547) [54], [55]. The trypsin gene (accession No: EU325547) in *H. armigera* was constitutively expressed, but its expression was influenced by 20-hydroxyecdysone (20E) and the juvenile hormone analog methoprene. This trypsin homolog had activity in the insect cuticle during larval feeding, molting and metamorphosis [55]. The authors suggest that this enzyme is involved in the remodeling of cuticle, but it is unlikely that orthologs in *O. nubilalis* (OnTry12 and OnTry13) have similar function as they lack residues critical for activity.

OnCTP15 and OnCTP16 were also predicted as chymotrypsin homologs, and were associated with orthologs from *M. sexta* (accession No: AM419170 and AM690448), *H. armigera* (accession No: EU325548), and *S. exigua* (accession No: AY820894) based on sequence analysis. In the *S. exigua* sequence, SeC34 (accession No: AY820894), the conserved motif ‘DSGGP’ in the catalytic domain is changed to ‘DSGSA’, and the enzyme was not inhibited by the chymotrypsin inhibitor TPCK. This gene was specifically expressed in the midgut of last instar larvae prior to the onset of pupation [56]. Another chymotrypsin from *H. armigera* (accession No: EU325548) has a similar sequence and activity properties and was associated with insect gut peritrophic matrix formation [54]. In this study, OnCTP15 and OnCTP16 have the same substitutions on the active site (DSGGP were replaced by DSDSA and DSGSA, respectively), but because they are classified as homologs, we are not certain if they are active enzymes.

### Diversity of Trypsins and Chymotrypsins in Lepidopteran Insects

The presence of multiple protease genes can be traced back to multi-copy genes that arose due to gene duplication and subsequent diversification events [57]. Mutation events in gene sequences may lead to amino acid alterations that influence the structural and functional properties of proteases, although these functional differences in insects are not fully understood. Host plants produce PIs to suppress insect proteinase activity as a defense mechanism against insect feeding. However, lepidopteran trypsins evidently have evolved to hydrolyze this kind of protein inhibitor and thereby avoid inhibition [15]. Insects may also overcome the negative effects of plant inhibitors by expressing inhibitor-insensitive proteases and/or up-regulating of other proteases [13], [14], [15], [58]. Jongsma and Bolter [32] emphasized the co-evolution of plants and herbivores, including plants expressing PIs as a defense against insects, and the well-adapted insect species that overcome the effects of such plant inhibitors. Therefore, the presence of multiple, varying protease-encoding genes in many lepidopteran species appears to be an adaption to diverse food sources and an adaptive mechanism for reducing the deleterious effects of plant PIs.

Trypsins from *H. armigera, S. nonagrioides*, and *H. zea* were identified and divided into three major groups based on their sensitivities to soybean PIs and substrate preferences [23], [58], [59]. In *M. configurata*, more than 30 trypsin and chymotrypsin or homolog transcripts, including eight trypsins, nine chymotrypsins, and 13 homologs were identified [60]. Similarly, in this study, we identified 34 trypsin, chymotrypsin and homolog transcripts, encoding 12 putative trypsins, 12 putative chymotrypsins, and 10 trypsin and chymotrypsin homologs from an *O. nubilalis* gut specific cDNA library. Most trypsins from lepidopteran insects were found in the Try-G2 and Try-G3 classification groups. However, all putative chymotrypsins were found in the large group CTP-G1. One chymotrypsin gene (*SlCTLP*) from *S. litura* (accession No. GQ354838) was food-inducible and was suppressed by starvation [26]. Therefore, the OnCTPs from CTP-G1 may play a similar role in protein digestion. The existence of multiple trypsins, chymotrypsins and homologs in *O. nubilalis* may be an adaptation to different food sources, as was described in *S. frugiperda*, a polyphagous insect, feeding on at least 80 species in 23 plant families [61]. *O. nubilalis* not only infests corn, but also uses potato and sweet pepper as temporary or alternative hosts [62], [63]. The alternative hosts can express different PIs, but *O. nubilalis* may express alternative serine proteases that are resistant. For example, *O. nubilalis* can overcome the effects of chicken ovomucoid, corn trypsin inhibitor, and potato protease inhibitor in artificial diets [64]. A previous study also found that Try-G2 trypsins from *H. armigera* are insensitive to Kunitz-type soybean trypsin inhibitor, whereas Try-G3 trypsins from *H. armigera* are sensitive to Kunitz-type soybean trypsin inhibitor [58], [59]. The data suggests that the diversity of trypsins is an adaptation against plant PIs and therefore an adaptation to diverse food sources.

### Tissue-Dependent Gene Expression Profiles

Thirty-four trypsin, chymotrypsin and homologous transcripts had slightly different expression patterns in the eight different tested tissues. As expected, all were highly expressed in midgut tissues, suggesting an important function in dietary protein digestion [65], [66]. Fifteen putative trypsins and trypsin homologs of *O. nubilalis* were found in two major groups, and trypsins of Try-G2 were highly expressed in the foregut and midgut, whereas those in Try-G3 were highly expressed in midgut and hindgut. The differential expression patterns imply that these putative trypsin and homolog genes may have different functions in food digestion. The tissue specific expression patterns were further supported by their sensitivity or insensitivity to Kunitz-type soybean trypsin inhibitor [58]. Among the trypsins of *A. ipsilon*, *H. armigera*, and *H. zea*, those in Try-G1 and Try-G3 were sensitive to the PI, whereas trypsins in Try-G2 were insensitive to PIs [13], [29], [59]. The foregut-midgut (Try-G2) trypsins may have developed insensitivity to PIs because they are the first to access and digest food sources containing PIs, thereby protecting the midgut-hindgut (Try-G3) trypsins, which are PI sensitive. It may also be possible that foregut-midgut trypsins play roles in activation of other zymogens, like trypsinogen, chymotrypsinogen and proelastase [67], or in initiating a cascade of events that subsequently regulate late trypsin expression [68]. Indeed, in *Aedes aegypti* adult female mosquitoes, multiple trypsin transcripts are sequentially induced with succeeding feeding periods, and an early trypsin may play a role in initiating a cascade of events with the subsequent expression of late trypsins [69], [70]. The foregut-midgut specific trypsins of *O. nubilalis* may have the similar roles.

We also found that *OnTry1* and *OnTry8* were slightly expressed in silk glands, so they may function in silk production [71]. *OnTry1* and *OnTry21* were expressed in all tested tissues, suggesting that they may be involved in many other important physiological functions, such as insect molting [72].

### Roles of Trypsins and Chymotrypsins in Bt Toxicity

The adaptation of *O. nubilalis* to anti-nutrients in plants may have inadvertently provided the insect an advantage that allows survival when exposed to sub-lethal concentrations of Cry toxins from transgenic corn. In this study, four putative trypsin genes (*OnTry4*, *OnTry5*, *OnTry6* and *OnTry14*) were up-regulated in *O. nubilalis* larvae, whereas one trypsin gene (*OnTry2*) was down-regulated at all time points after the ingestion of Cry1Ab protoxin. Down-regulated serine proteases may be implicated in Bt resistance if they are involved in the solubilization and activation of Bt protoxins [5]. Several studies have shown that trypsins hydrolyze Bt protoxins or toxins, and have been genetically linked to Bt resistance [5], [37], [38], [56]. In fact, the first example of protease-mediated resistance to Bt protoxins was in Bt-resistant *P. interpunctella* lacking a major trypsin enzyme [5]. Midgut enzymes from a Bt resistant strain of *H. virescens* were reported to activate the protoxin slower but to degrade the toxin faster than the enzymes from a susceptible strain [73], [74]. Another study also found that when Bt protoxin was used in mosquito control, trypsin was the major gut enzyme that activated the 130-kDa Cry4B protoxin into a 65-kDa toxin [75]. However, activated toxin was able to efficiently control mosquito larvae because it was resistant to hydrolysis by midgut proteases. In our lab, the soluble trypsin-like proteinase activity of a resistant *O. nubilalis* strain was approximately half that of a susceptible strain [37]. Reduced trypsin-like activity was attributed to reduced expression of OnT23 in Bt-resistant *O. nubilalis* [38]. In *S. frugiperda*, knockdown of a trypsin SfT6 (accession No: FJ940726) reduced susceptibility to Cry1Ca1 [28]. Our phylogenetic analysis indicated that OnTry4, OnTry5, OnTry6 and OnTry14 in *O. nubilalis*, up-regulated after 6 h Cry1Ab protoxin exposure, shared 68–79% amino acid identity with SfT6 and are in the same group (Try-G2).

Chymotrypsin has been demonstrated to be involved in toxin degradation in some insects. For example, the chymotrypsin inhibitors (e.g., TPCK) showed strong inhibitory effects against further degradation of activated toxin from *B. thuringiensis* subsp. *kurstaki* HD-1 in *H. armigera* midgut juice [76]. This result indicates that chymotrypsin plays a major role in degradation of Bt toxin. In our study, we found that three putative chymotrypsins transcripts (*OnCTP2*, *OnCTP5*, and *OnCTP12*) and one chymotrypsin homolog (*OnCTP13*) were significantly up-regulated after a 24-h exposure to Cry1Ab in *O. nubilalis*. Furthermore, chymotrypsin up-regulation (after 24 h exposure) was much later than trypsin up-regulation (2 to 6 h exposure). It is possible that the chymotrypsins encoded by those transcripts may contribute to the degradation of activated Cry1Ab toxin in *O. nubilalis*, especially OnCTP2 and OnCTP12, which were up-regulated more than 10 fold after 24 h exposure to Cry1Ab. The increased expression of chymotrypsin transcripts has been noted in coleopteran responses to Cry3Aa [77] as well as PIs [53].

Although our analysis of the transcriptional response of the trypsin and chymotrypsin genes in response to Cry1Ab in the gut of *O. nubilalis* larvae was carried out after relatively short periods (2, 6, 12 and 24 h) of larval feeding, possible effects of the protoxin on general physiological processes may have already occurred, particularly at the late time point (i.e., 24 h). Thus, transcriptional changes in some of these genes may not necessarily indicate the involvement of these genes in Cry1Ab intoxication or detoxification. Indeed, Oppert et al. [78] found a complex transcriptional response to Cry3Aa in yellow mealworm (*Tenebrio molitor*) larvae during the initial 24 h of intoxication. The exposures of *T. molitor* larvae to Cry3Aa resulted in an induction of genes involved in mitochondrial electron transport, signaling, carbohydrate metabolism, membrane components, cell structure, and allergens, but caused a repression of genes encoding metabolic enzymes associated with proteolysis, glycolysis, TCA, and fatty acid metabolism. Furthermore, Oppert et al. [77] also found some significant effect of Cry3Aa on the expression of serine protease transcripts potentially involved in protoxin processing. Thus, although our study provided the first insights into transcriptional responses of these putative trypsin and chymotrypsin genes to the ingestion of Cry1Ab protoxin, further research is needed to clarify the role of these transcripts in intoxication (e.g., activation of the protoxin) or detoxification (e.g., degradation of the protoxin or toxin) of Cry1Ab in *O. nubilalis* larvae.
